# Effect of Nitrogen Cation as “Electron Trap” at π-Linker on Properties for *p*-Type Photosensitizers: DFT Study

**DOI:** 10.3390/molecules24173134

**Published:** 2019-08-28

**Authors:** Zhi-Dan Sun, Jiang-Shan Zhao, Xue-Hai Ju, Qi-Ying Xia

**Affiliations:** 1 Key Laboratory of Soft Chemistry and Functional Materials of MOE, School of Chemical Engineering, Nanjing University of Science and Technology, Nanjing 210094, China; 2 School of Chemistry and Chemical Engineering, Linyi University, Linyi 276005, China

**Keywords:** *p*-type dye-sensitized solar cells (DSSCs), dyes, π-linker, amine salt, density functional theory (DFT)

## Abstract

On the basis of thieno(3,2-*b*)thiophene and dithieno[3,2-*b*:2′,3′-*d*]thiophene (**T2** and **T3** moieties) as π-linker, the **A**, **D** and **S** series dyes were designed to investigate the effect of the introducing N^+^ as an “electron trap” into **T2** and **T3** on the properties of the dyes. The optimized structures, electronic and optical properties were investigated by the density functional theory (DFT) and time-dependent DFT (TD-DFT). The results show that the properties of the dyes are sensitive to the N^+^ position in π-linkers. **D** series dyes with electron-withdrawing units located near the donor have better properties than the corresponding **A** series with the electron-withdrawing units located near the acceptor. For **A** and **D** series, the N^+^ modified dye named **T2N+1-d** displays the largest red shift of the UV–vis absorption, the maximum integral values of the adsorption-wavelength curves over the visible light, the highest light harvesting efficiency (LHE, 0.996), and the strongest adsorption energy (−44.33 kcal/mol). **T2N+1-d** also has a large driving force of hole injection (Δ*G*_inj_, −0.74 eV), which results in a more efficient hole injection. Bearing a lengthier π-linker than **T2N+1-d**, the properties of **T2N+1-s** are further improved. **T2N+1-d** moiety or its increased conjugated derivatives may be a promising π-linker.

## 1. Introduction

Dye-sensitized solar cells (DSSCs) have attracted considerable attention, owing to their great potential of being environment-friendly and low-cost, ever since crucial contributions were made by Grätzel and co-workers in 1991 [1]. However, the development of tandem dye-sensitized solar cells (DSSCs) is limited by the low efficiency of *p*-type DSSCs [2,3]. For future applications, more studies on *p*-type DSSCs are needed.

In the design of a donor-acceptor dye, the π-linker group between the donor and the acceptor plays an important role, and thus has been widely studied [4,5,6,7]. Electron-rich thiophene (**T1** moiety) was widely served as a π-linker for DSSCs due to its good performance for charge–transfer interaction and photovoltaic properties [8]. Therefore, many dyes with thiophene derivatives as π-linkers have been synthesized and investigated [5,9]. N atoms are often used to modify the dyes to improve the photovoltaic properties of DSSCs [10,11,12]. For organic heterocycles containing an N atom, lone pair electrons on amine can undergo an alkylation reaction to form amine salt (N^+^) [13,14]. The amine salt groups with strong electron-withdrawing ability can be used as the “electron trap” to modify the dyes [15]. The suitable introduction of electron-withdrawing units into the π bridge as an “electron trap” can improve the distribution of donor electrons and facilitate electron transfer from the donor to the anchor, thereby improving the photovoltaic properties of the dyes [3]. Therefore, for *p*-type DSSCs, N^+^ modified thiophene derivatives as π-linkers may improve the performance of *p*-type dyes, and the location of N^+^ in the π-linker also has an important influence on the performance of dyes. The dyes with N^+^ moiety in the conjugation path, acting as *p*-type photosensitizers, have been synthesized [3,16]. However, the effect of N^+^ on the properties for *p*-type sensitizers at different positions of the π-linker has rarely been reported

The synthesis of new dyes is time consuming, thus, theoretical study is considered as a highly efficient way to investigate the relationship between the molecular structures and the chemical properties of dyes. The rational design of sensitizers is an important way to improve dye performance. In addition, DFT and TD-DFT methods are widely employed to optimize the geometrical structures and to evaluate absorption spectra and molecular performance of the sensitizers in DSSCs.

**Figure 1:**
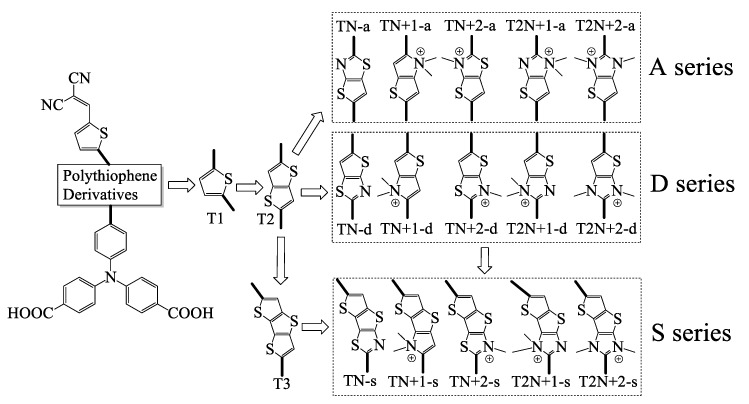
Molecular structures of the investigated dyes.

The dyes with thieno(3,2-*b*)thiophene and dithieno [3,2-*b*:2′,3′-*d*]thiophene (**T2** and **T3** moieties) as π-linkers have been proved to improve the performance of DSSCs [15,17]. In this study, we chose triphenylamine as an electron donor with the attachment of two –COOH as anchoring groups and dicyanovinyl as an acceptor, which are the most widely selected in both synthesis and theoretical studies of *p*-type DSSCs [4,18]. Furthermore, the **A**, **D,** and **S** series of dyes were designed to investigate the effect of the introducing N^+^ as an “electron trap” into **T2** and **T3** on the properties of the dyes. The molecular structures of the investigated dyes are listed in Figure 1. The properties of the dyes are calculated by DFT and TD-DFT methods. We expected that this study could shed some light on the molecular design of *p*-type photosensitizers.

## 2. Results and Discussion

### 2.1. UV–Vis Absorption Spectra

**Figure 2:**
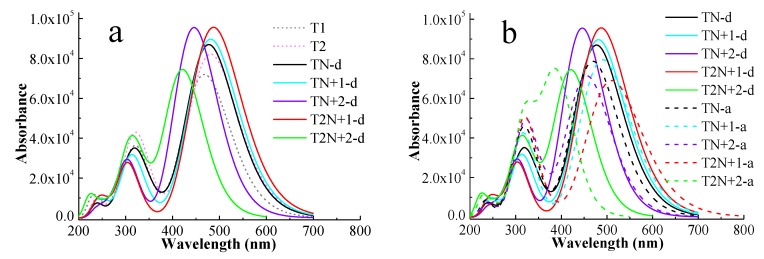

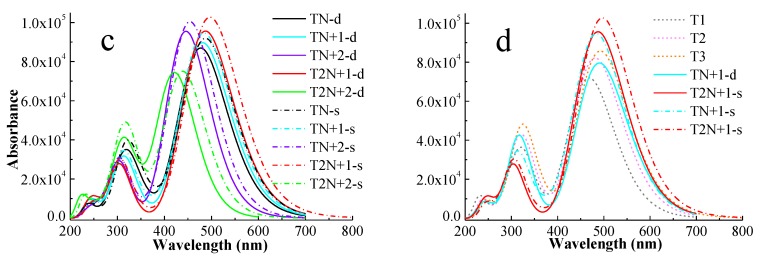
Absorption spectra of dyes. Comparison of T1, T2, and D series (**a**), comparison between D and A series (**b**), comparison between D and S series (**c**), and comparison of the selected dyes and T series (**d**).

UV–vis absorption spectrum is an important characteristic for evaluating the properties of sensitizers for DSSCs. A high efficiency sensitizer should have a broad and strong absorption over the visible light (400~800 nm). In order to ensure the reliability of the calculation, we simulated the UV–vis absorption spectrum of **O2** at the CAM-B3LYP/6-311G** level with a continuum solvation model in acetonitrile by using the Gaussian 09 program. **O2** has been synthesized and has a similar structure to **T1**. As shown in Figure S1, the maximum absorption wavelength and the half-width of the Gaussian band for the simulated UV–vis absorption spectrum of **O2** are close to the experimental results [4]. Therefore, the simulated UV–vis absorption spectra for all dyes in this study were calculated at the same level, which are displayed in Figure 2. Figure 2a is the spectra of **T1**, **T2,** and **D** series dyes. As shown in Figure 2a, **T2** has a red shift of the UV–vis absorption and an enhanced absorption of visible light in comparison with **T1** due to an increased length of π-linker. Compared with **T2**, the intensities of the maximum absorption peaks of **D** series dyes are enhanced except **T2N+2-d**, and the maximum absorption wavelengths of **D** series dyes are red or blue shifted. For **TN-d**, the maximum absorption wavelength changes little, and the intensity of it is enhanced in comparison with **T2**, which is related to the *p*-electrons on the N atom of the π-linker promoting the intramolecular charge transfer of the dye [19]. For the other four **D** series dyes with π-linkers containing N^+^, the maximum absorption wavelengths of **TN+1-d** and **T2N+1-d** show red shifts, while those of **TN+2-d** and **T2N+2-d** show blue shifts. This is attributed to the different locations of N^+^ on the π-linkers. The suitable introduction of electron-withdrawing units into the π bridge can improve the photovoltaic properties of the dyes [20,21,22]. For **D** series dyes, the introduction of N^+^ near the donor act as an “electron trap” and can facilitate the intramolecular charge transfer from donor to acceptor, so **T2N+1-d** has a red shift and an increasing absorption in comparison with **TN-d**. Compared with **T2N+1-d**, there is no electron-rich N atom in the π-linker of **TN+1-d**, so the intramolecular charge transfer is weakened. Therefore, the UV–vis absorption spectrum of **TN+1-d** has an obvious blue shift with decreasing absorption strength in comparison with **T2N+1-d**, but has a small red shift and absorption enhancement in comparison with **TN-d** due to the introduction of N^+^. For **TN+2-d** and **T2N+2-d**, the locations of N^+^ are on double bond of π-linkers, which is the main path of intramolecular charge transfer [23]. This can prevent the intramolecular charge transfer somewhat due to the strong electron-withdrawing ability of N^+^. In addition, thiophene has good aromaticity and π electron delocalization, thus the UV–vis absorption spectrum of **TN+2-d** containing N^+^ has an absorption enhancement and a blue shift in comparison with **TN-d**. For **T2N+2-d**, the aromaticity was reduced due to the substitution of the S atom with the N atom in the π-linker, and the extra methyl group on N atom will make the molecular less co-planar. Hence, the UV–vis absorption spectrum of **T2N+2-d** has the most blue shift and the weakest absorption in **D** series dyes.

Figure 2b shows **D** and **A** series dyes. The full width at half maximum (FWHM) of the strongest absorption peak for all dyes are displayed in Table S1. As can be seen in Figure 2b, the UV–vis absorption spectra of **D** series dyes have a stronger absorption than that of the corresponding **A** series dyes. The difference of the structure between **D** and **A** series dyes is that the N^+^ positions of the dyes in **D** series are located near the donor, and those of the corresponding **A** series dyes are near the acceptor. Impressively, the stronger the intensity of the maximum absorption peak of **D** series is, the weaker the intensity of its corresponding **A** series is. Similar to many D-A-π-A structural dyes, the suitable introduction of N^+^ in the π-linker near the donor act as an “electron trap” that can improve the distribution of donor electrons and facilitate electron transfer from the donor to the anchor, as shown in Figure 2a. However, as the distance between N^+^ and donor increases, the effect of the electron-withdrawing ability for N^+^ on the donor decreases and the effect on the acceptor increases. N^+^ in π-linkers for **A** series is located near the acceptor, and the strong electron-withdrawing ability of N^+^ can significantly hinder the electron transfer towards the acceptor and hardly affect the distribution of donor electrons. Therefore, unlike **D** series dyes, the photovoltaic properties of **A** series dyes become worse.

Figure 2c shows the absorption spectra of **D** and **S** series dyes. By increasing the lengths of the π-linkers, the dyes in the **S** series display red shifts and UV–vis absorption enhancement in comparison with the corresponding ones in **D** series dyes. In addition, the shifts of UV–vis absorption for the **S** series are similar to the **D** series and the UV–vis absorption spectra of **T2N+1-d** and **T2N+1** dyes are the best in the **D** and **S** series, respectively. As shown in Figure 2d and Table S1, although the π-linker length of **T3** is longer than that of **T2N+1-d**, **T2N+1-d** displays an obviously broader and stronger UV–vis absorption than **T3**. For **TN+1-s** and **T2N+1-s**, which have the similar lengths of the π-linkers of **T3**, the UV–vis absorption spectra display much stronger UV–vis absorption than that of **T3**.

**Figure 3:**
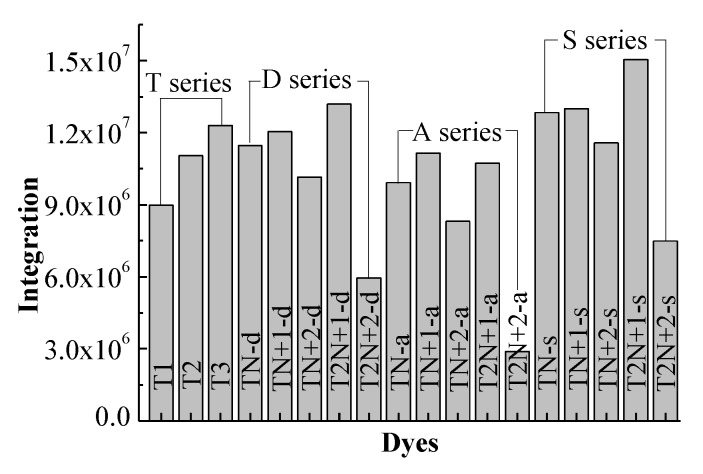
The integral area of absorption-wavelength curve over 400~800 nm.

Figure 3 shows the integral area of the absorption-wavelength curve over 400~800 nm. for all dyes. The integral values of the absorption-wavelength curves for **D** series dyes are larger than the corresponding **A** series dyes. The introduction of N^+^ as an “electron trap” in the π-linker near the donor is beneficial to sunlight absorption. The order of the integral areas of the absorption curves over 400~800 nm for **D** series dyes is **T2N+1-d** > **TN+1-d** > **TN-d** > **TN+2-d** > **T2N+2-d**. **T2N+1-d** has the largest integral area in the **D** series, which is greatly improved in comparison with **T2**. Those rules are the same for **S** series dyes. The onset at half maximum of the strongest absorption peak is an important factor. The onset values of all dyes are listed in Table S1. As shown in Table S1, the onset values of **T2N+1-d** and **T2N+1-s** dyes are the largest in the **D** and **S** series, respectively. The two dyes have the minimum necessary excitation energy. Therefore, the suitable introduction of N^+^ in dyes can regulate the absorption of visible light. The dyes with **T2N+1-d** moiety and lengthier π-linker derivatives may improve the photovoltaic properties for *p*-type DSSCs more effectively.

### 2.2. Electronic Structures of Dyes

**Figure 4:**
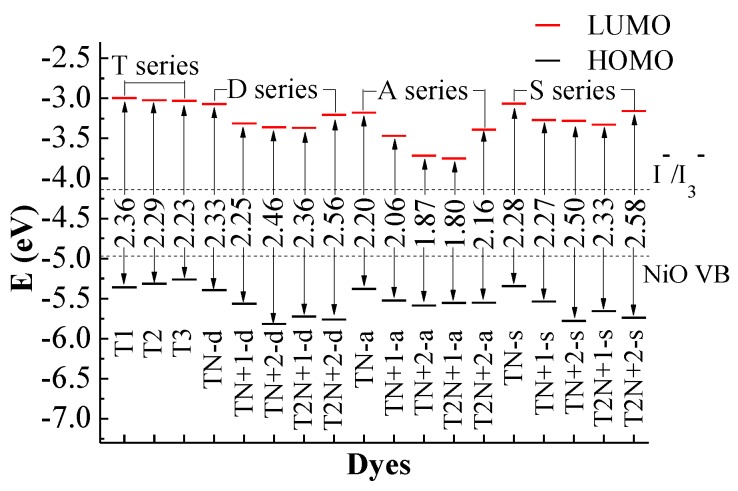
Frontier molecular orbital energy levels and energy gap, together with *E*_VB_ (NiO) and *E*(I^−^/I_3_^−^).

In order to ensure a fast and efficient hole transfer and separation in the *p*-type DSSCs, the potential levels of the HOMO must be lower than the NiO valence band and the LUMO must be higher than I^−^/I_3_^−^ redox potential [24,25]. Figure 4 shows the frontier molecular orbital energy levels from the (U)B3LYP/6-31G** calculations for all dyes, together with the experimental energy of the valence band of semiconductor NiO (*E*_VB_, −4.98 eV) and the redox potential of the mediator *E*(I^−^/I_3_^−^, −4.15 eV) [5,26]. As shown in Figure 4, the LUMO levels of all dyes are above the I^−^/I_3_^−^ redox couple and the HOMO levels of all dyes are below the NiO valence band (VB), which matches well with the NiO semiconductor electrode and I^−^/I_3_^−^ electrolyte. Hence, all the dyes in this study can be used as dye sensitizers for the *p*-type DSSCs.

For **T** series dyes, the HOMOs are close to the VB of NiO. This is not beneficial to the hole injection from the excited dye to the semiconductor. For *p*-type DSSCs dyes, the efficient hole injection is one of the most important factors for the performance of dyes [27]. Thus, the lower HOMO level is very important for *p*-type dyes. For **D** series dyes, especially the four dyes containing N^+^, the energy levels of HOMO and LUMO move towards a more negative potential in comparison with **T2**. For the four dyes containing N^+^ of the **D** series, the HOMOs locate well below the NiO valence band, and the LUMOs locate well above the I^−^/I_3_^−^ redox potential. The variation of the HOMO and LUMO levels for **S** series dyes is similar to **D** series dyes. Therefore, the suitable introduction of N^+^ in dyes can regulate the HOMO and LUMO levels and improve the hole injection. Compared with **T2**, the HOMO levels of the dyes containing N^+^ in the **A** series move towards negative potential slightly, while the LUMO levels greatly move towards negative potential. This is because the electron-withdrawing N^+^ is too close to the acceptor of the dyes, and the strong electron-withdrawing ability lowers the LUMO levels of the dyes significantly. This reduces the dye regeneration.

The HOMO-LUMO energy gaps of all dyes are also displayed in Figure 4. The energy gaps of **A** series dyes are smaller than those of the other series. This is because the electron-withdrawing unit N^+^ for **A** series dyes is close to the acceptor of the dyes, which lowers the LUMO levels and hinders the electron transfer towards the acceptor. Therefore, even if the energy gaps of **A** series dyes are smaller, the sunlight absorptions are still weaken (Figure 2). For **D** series dyes, compared with **T2**, the energy gaps of **TN+2-d** and **T2N+2-d** increase, and those of **TN-d**, **TN+1-d** and **T2N+1-d** change little. This is attributed to the effect of the different locations of N^+^. For **TN+2-d** and **T2N+2-d**, the locations of N^+^ are on the double bond of the π-linkers and the effect of N^+^ on the charge transfer is stronger, so that the energy for the charge transfer increases. The variations of the energy gaps for **S** series dyes are similar to those of **D** series. The energy gaps of **T2N+1-d** and **T2N+1-s** are 2.36 and 2.33 eV, respectively. Compared with **T** series dyes, the values of energy gaps for **T2N+1-d** and **T2N+1-s** change little, but the levels of HOMO and LUMO are effectively improved. The onset at half maximum of the strongest absorption peak has an effect on the energy gap of dyes. In general, the large onset value will lead to a small energy gap, but many other factors also affect the value of the energy gaps such as a large conjugated region of π-linker, an introduction of an electron withdrawing group and so on. Therefore, the onset values of **T2N+1-d** and **T2N+1-s** dyes are the largest in **D** and **S** series, respectively, but the energy gaps of them are not the smallest.

The distributions of molecular frontier orbitals are displayed in Table 1. As can be seen in Table 1, the HOMOs distribute mainly on the “D-π-” units in “D-π-A” system, while LUMOs dominantly on the “-π-A” units. The dyes can smoothly delivery the electrons from the donors to the acceptors. Importantly, for the dyes containing N^+^ in the π-linker, there is less HOMO distribution on the acceptor direction than the dyes without N^+^. The less HOMO distribution on the acceptor direction is favorable for hole injection for *p*-type DSSCs. Therefore, the introduction of N^+^ in π-linkers is beneficial to improve the distributions of molecular frontier orbitals.

### 2.3. Performances of p-Type Sensitizers

It is well known that the energy conversion efficiency (*η*) is closely related to the short-circuit photocurrent density (*J*_sc_) and the open-circuit photovoltage (*V*_oc_) [28]. As the electrode is same, the *J*_sc_ was closely affected by the light-harvesting efficiency (LHE) and three vital parameters: The driving force of hole injection (Δ*G*_inj_) from the excited dye to the semiconductor, the driving force of regeneration (Δ*G*_reg_) between the oxidized dye and the electrolyte, and the driving force of charge recombination (Δ*G*_CR_) from the oxidized dye to the semiconductor [3,29]. The more negative Δ*G*_inj_ and Δ*G*_reg_ will be beneficial for hole injection and dye regeneration [11]. However, the more negative Δ*G*_CR_ will cause the charge recombination to easily occur, to a certain extent [11]. For *p*-type DSSCs dyes, efficient hole injection is one of the most important factors affecting the performance of dyes because of the electron-withdrawing anchor located in the donor groups [27]. Therefore, Δ*G*_inj_ is more important for *p*-type dye.

The computed ∆*G*_inj_, ∆*G*_reg_, ∆*G*_CR_, transition configuration and LHE of the dyes are listed in Table 2. As can be seen in Table 2, the dyes of **D**, **A** and **S** series have more negative ∆*G*_inj_ than **T** series dyes, especially the dyes containing N^+^. This indicates that the introduction of N^+^ in π-linkers can effectively improve the hole injection for *p*-type DSSCs. In addition, ∆*G*_inj_ of **D** and **S** series containing N^+^ is more negative than the corresponding one of **A** series dyes. Hence, the introduction of N^+^ in π-linkers near the donor of dyes is more beneficial to the hole injection. Compared with **T** series dyes, both ∆*G*_reg_ and ∆*G*_CR_ of **D**, **A**, and **S** series become less negative. The variation of ∆*G*_CR_ could suppress the charge recombination to some extent and that is favorable for the performance of dyes. The variation of ∆*G*_reg_ is not beneficial for dye regeneration, but the values of ∆*G*_reg_ for the **D** and **S** series change not so much and Δ*G*_inj_ is more important factor for the performance of *p*-type dyes. Therefore, the introduction of N^+^ in π-linkers near the donor can improve the performance of *p*-type dyes. The Δ*G*_inj_ of **TN+2-d**, **T2N+1-d**, and **T2N+2-d** are −0.84, −0.78, and −0.76 eV, respectively, which were significantly improved in comparison with the **T** series. For the **S** series, the order of the Δ*G*_inj_ values is consistent with that of **D** series dyes, but the Δ*G*_inj_ values become slightly less negative due to an increased length of π-linker.

According to the transition configurations displayed in Table 2, the largest portion of transition configuration is HOMO to LUMO, and the HOMO−1 and LUMO+1 also take part in the transition. A larger portion of HOMO to LUMO taking part in the transition is beneficial for the hole injection from the dye to the valance band of NiO [30]. The contributions of HOMO to LUMO transition for **T2N+1-d** and **T2N+1-s** are 74% and 77%, which are the largest in **D** and **S** series, respectively, and are improved in comparison with **T** series dyes. The contributions of HOMO to LUMO transition for **T2N+2-d** and **T2N+2-s** are 28% and 43%, which are the lowest in **D** and **S** series, respectively, and lower than those of the **T** series. For other dyes of the **D** and **S** series, the contributions of HOMO to LUMO transition are similar to those of the **T** series. However, the contributions of HOMO to LUMO transition for the **A** series are all smaller than those of the **T** series.

The LHE is closely to the oscillator strength (*f*) of dye and can be approximated as [5]

(1) $$\mathrm{LHE}\approx 1-10^{-f}$$

As shown in Table 2, all the dyes of **D** series except **T2N+2-d** have higher LHE than **T2**, while all the dyes of **A** series have lower LHE than **T2**. For the **S** series, the LHE are further improved by increasing the π-linkers. The LHE of **T2N+1-d** is 0.996, which is the best in the **D** series. For the **S** series, the LHE of **T2N+1-s** is also the best. Therefore, the suitable introduction of N^+^ in π-linkers near the donor can improve the transition contributions and LHE of dyes.

The introduction of **T2N+1-d** moiety as a π-linker into the dyes can improve the Δ*G*_inj_ and ∆*G*_CR_, enhance the HOMO to LUMO transition and increase the LHE, and thus improve the performance of *p*-type DSSCs. Compared to **T2N+1-d**, the performance of **T2N+1-s** with an increased length of π-linker will be further improved.

### 2.4. Effect of the Counterion of N^+^ Moiety on the Photophysical Properties of Dyes

**Figure 5:**
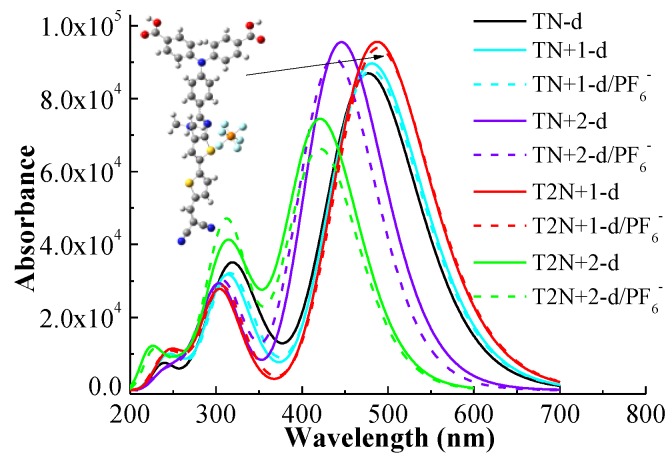
The comparison of the simulated UV-vis absorption spectra between RN^+^/PF_6_^−^ and the RN^+^.

The dyes with N^+^ moiety act as *p*-type photosensitizers have been synthesized and reported in the literatures [3,16,31]. Indeed, there could be an interaction between N^+^ moiety of the dyes and the counterions. In the study of Marri et. al, a series of amine salt containing methylpyridine cation and PF_6_^−^ anion is synthesized to be used as *p*-type photosensitizers [31]. Here PF_6_^−^ anion was chosen as the counterion of N^+^ moiety (RN^+^) to investigate its effect on the photophysical properties of dyes. The RN^+^/PF_6_^−^ geometrical structures of **D** series dyes and their UV-vis absorption spectra were calculated at the same level as N^+^ cation. Figure 5 shows the comparison of the simulated UV-vis absorption spectra between RN^+^/PF_6_^−^ and RN^+^. Compared with the UV-vis absorption spectra of RN^+^, the wavelengths of the absorption peaks for RN^+^/PF_6_^−^ hardly change, and the intensities of the maximum absorption peaks change little. Importantly, the conclusions obtained from **D** series dyes are consistent with that of RN^+^/PF_6_^−^. For the four dyes containing N^+^ in **D** series, the absorption spectra of **TN+1-d** and **T2N+1-d** are hardly affected by PF_6_^−^ anion, but the intensities of the maximum absorption peaks of **TN+2-d** and **T2N+2-d** are weakened a little by PF_6_^−^ anion. This is related to two methyl groups connected to the N^+^ for **TN+1-d** and **T2N+1-d**, which can better protect N^+^ in the conjugation ring from the electronegative groups. For **TN+2-d** and **T2N+2-d**, there is only one methyl connected to the N^+^, which is coplanar with the conjugation ring where N^+^ locates in. This may lead to the electronegative groups approaching N^+^ less difficultly and the interaction between the N^+^ moiety and the electronegative groups enhanced. Hence, the intramolecular charge transfer toward acceptor for **TN+2-d** and **T2N+2-d** can be hindered somewhat by the electronegative groups, and the intensities of the maximum absorption peaks are weakened.

**Figure 6:**
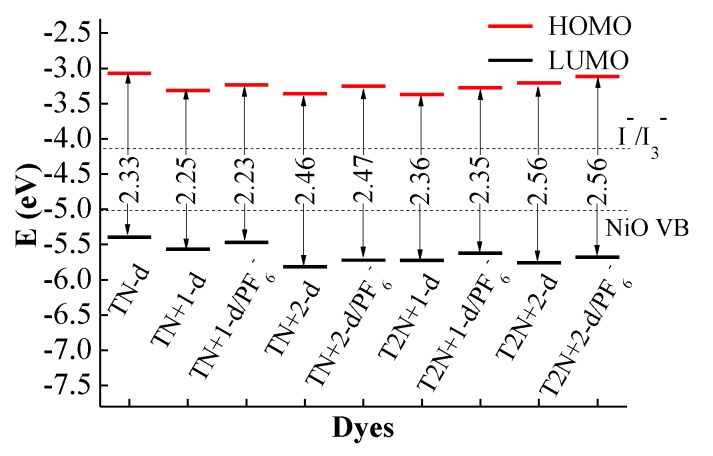
Comparison of the frontier molecular orbital energy levels and energy gaps of RN^+^/PF_6_^−^ and the RN^+^.

The comparison of the frontier molecular orbital energy levels and energy gap between RN^+^/PF_6_^−^ and the RN^+^ are shown in Figure 6. Compared with the RN^+^, the energy levels of HOMO and LUMO for the RN^+^/PF_6_^−^ systems move towards a more positive potential, but the changes are very little. The values of the energy gap for the RN^+^/PF_6_^−^ systems are almost as the same as those of the RN^+^.

Therefore, the effect of the counterion of N^+^ moiety on the photophysical properties of dyes is very little. The conclusions obtained from **D** series dyes are consistent with that of RN^+^/PF_6_^−^ systems. The properties calculated by the single structure of the dyes containing N^+^ are reliable and highly efficient.

### 2.5. The Dye/NiO Interaction

In DSSCs, the interaction between dye and the semiconductor interface plays a crucial role on hole injection efficiency [26]. Generally, the value of adsorption energy of the dye/NiO system indicates the strength of the interaction between the dye and the NiO surface. A larger adsorption energy will lead to a stronger electronic coupling strength between the anchoring group of the dye and the NiO surface, which also increases the hole transfer rate [26]. To calculate the adsorption energies, a nickel oxide (NiO) cluster with two layers of 12 × 3 NiO were created, and then the optimized configuration of all ten dyes was located on the NiO surface, respectively. The bottom NiO layer of all the dye/NiO system was fixed, while other atoms were allowed to relax. Each structure of the dye/NiO system was optimized under a solvation model at the GGA-PBE/DN level, using the Dmol^3^ program.

**Figure 7:**
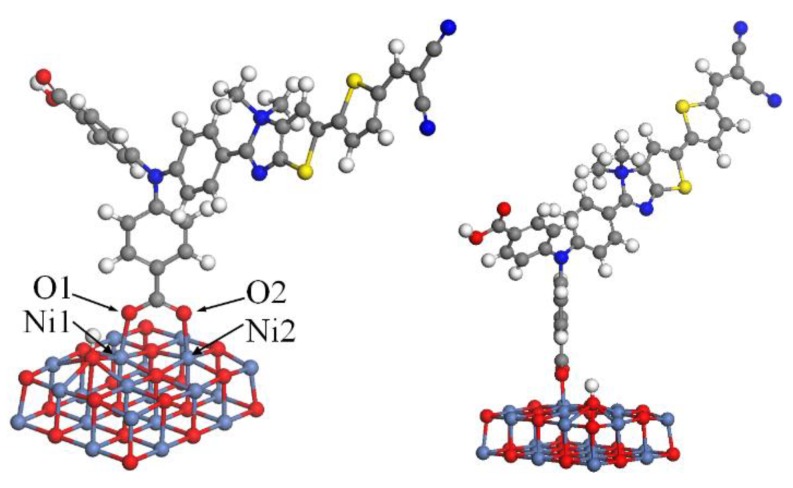
Adsorption of **T2N+1-d** on (NiO)_10__× 2_.

An example of the optimized dye/NiO structures with the lowest energy is presented in Figure 7, in which the dye binds onto the surface through the carboxyl group. As can be seen in Figure 5, the dye binds almost perpendicular to the NiO surface via bidentate coordination bridging that is stable [32,33]. The adsorption energies and the calculated bond lengths of Ni1–O1 and Ni2–O2 are also listed in Table 3. The Ni–O bond lengths of all dyes are in the range of 1.99 to 2.07 Å. These values are in good agreement with that from the literature [34].

As shown in Table 3, the adsorption energy values of all dyes are in the range of −37.88 to −45.22 kcal/mol. For **T** series dyes, the adsorption energy values increase in the following order **T1** < **T2** < **T3**. This indicates that increasing the length of the π-linker is beneficial to improving the adsorption energy to some extent. For **D** series dyes, the adsorptions are enhanced in comparison with **T2**, especially for the dyes containing N^+^. This indicates that the introduction of N^+^ in the π-linker can enhance the interaction between dyes and the NiO electrode. The adsorption energy values of the **D** series increase in the following order **TN-d** < **T2N+2-d** < **TN+1-d** < **TN+2-d** < **T2N+1-d**. The adsorption energy value of **T2N+1-d** is −44.33 kcal/mol, which is the largest in the **D** series. This will lead to a stronger adsorption on the semiconductor surface. The adsorption energy value of the **A** series is lower than the corresponding one of the **D** series. This is because the distance between the electron-withdrawing unit N^+^ of the **A** series and the NiO surface is longer than that of the **D** series, which makes the interaction between the **A** series and the NiO surface weaker. The adsorption energy of **T2N+1-a** is also the largest in the **A** series, which is similar to the **D** series. For **S** series dyes, the adsorption energy values of are larger than the corresponding **D** series due to the longer π-linkers of the **S** series. Interestingly, the order of adsorption energy values of **S** series dyes is consistent with that of the **D** series and the dye with the largest adsorption energy in the **S** series is **T2N+1-s**, too. Therefore, the introduction of **T2N+1-d** moiety as a π-linker into the dyes can enhance the interaction between the dyes and the NiO electrode more effectively, and an appropriate increase in the length of the π-linker will increase the adsorption energy.

## 3. Computational Methods

The molecular structure and electronic properties of all dyes were investigated using the quantum chemical program Gaussian 09 [35]. The geometrical structures of the molecules were optimized at the (U)B3LYP/6-31G** level, which was widely used for *p*-type triphenylamine system [36,37]. TD-DFT is an economical method of modeling excited states. Thus, the excited state properties were calculated at CAM-B3LYP/6-311G** level by the TD-DFT, which was widely applicable for predicting the properties of excited states [38,39]. Solvent effects were included by using acetonitrile as the solvent in the whole Gaussian calculation, and the polarized continuum model (PCM) was taken into account throughout. The structures of the dye/NiO system were optimized under a solvation model at the GGA-PBE/DN level, using the Dmol^3^ program of Materials Studio 6.0. The adsorption energies were calculated at the same level.

## 4. Conclusions

The **A**, **D** and **S** series dyes were designed to investigate the effect of the introduction of N^+^ as an “electron trap” on the properties of the dyes. The results show that all the dyes can be used as dye sensitizers for the *p*-type DSSCs and the properties of the dyes are sensitive to the N^+^ position in π-linkers. The introduction of N^+^ near the donor act as the “electron trap” and facilitate the intramolecular charge transfer from donor to acceptor, so the **D** series has better properties than the corresponding **A** series. The dye of **T2N+1-d** displays the largest red shift of the UV–vis absorption, the maximum integral values of the adsorption-wavelength curves over the visible light, the highest light harvesting efficiency (LHE, 0.996), and the strongest adsorption energy (−44.33 kcal/mol) on NiO surfaces in **D** series dyes. In addition, **T2N+1-d** has a large driving force of hole injection (Δ*G*_inj_, −0.74 eV), which results in a more efficient hole injection. By increasing the length of the π-linker of **T2N+1-d**, the properties of **T2N+1-s** are further improved. The suitable introduction of N^+^ can improve the performance of dyes, and the **T2N+1-d** moiety or its extended conjugation derivatives may be a promising π-linker for *p*-type DSSCs.

## Figures and Tables

**Table 1:** Contours of molecular frontier orbitals of dyes.

Dyes	HOMO	LUMO	Dyes	HOMO	LUMO
**T1**	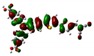	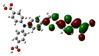	**T2**	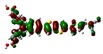	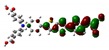
**T3**	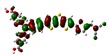	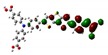	**TN-d**	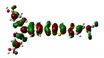	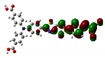
**TN+1-d**	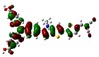	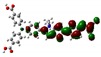	**TN+2-d**	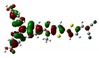	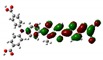
**T2N+1-d**	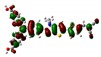	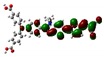	**T2N+2-d**	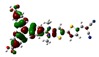	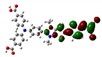
**TN-a**	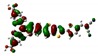	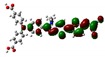	**TN+1-a**	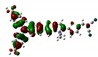	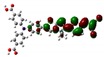
**TN+2-a**	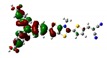	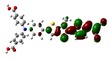	**T2N+1-a**	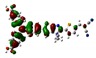	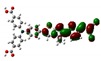
**T2N+2-a**	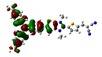	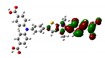	**TN-s**	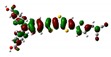	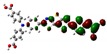
**TN+1-s**	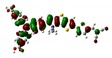	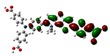	**TN+2-s**	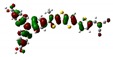	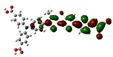
**T2N+1-s**	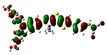	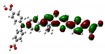	**T2N+2-s**	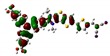	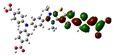

**Table 2:** Computed ∆*G*_inj_, ∆*G*_reg_, ∆*G*_CR_, transition configuration and LHE of the dyes.

Dyes	∆*G*_inj_ (eV) ^a^	∆*G*_reg_ (eV) ^b^	∆*G*_CR_ (eV) ^c^	*f*	Main Configurations	LHE
**T1**	−0.38	−1.15	−1.98	1.7761	H → L (69%), H − 1 → L (24%)	0.983
**T2**	−0.33	−1.13	−1.96	2.0281	H → L (70%), H − 1 → L (22%)	0.991
**T3**	−0.28	−1.12	−1.95	2.1153	H → L (71%), H − 1 → L (20%)	0.992
**TN-d**	−0.42	−1.08	−1.91	2.1453	H → L (69%), H − 1 → L (23%)	0.993
**TN+1-d**	−0.59	−0.84	−1.67	2.2128	H → L (65%), H − 1 → L (23%)	0.994
**TN+2-d**	−0.84	−0.79	−1.62	2.3563	H → L (54%), H − 1 → L (30%)	0.996
**T2N+1-d**	−0.74	−0.78	−1.61	2.3603	H → L (74%), H − 1 → L (13%)	0.996
**T2N+2-d**	−0.78	−0.95	−1.78	1.8304	H → L (28%), H − 1 → L (65%)	0.985
**TN-a**	−0.40	−0.97	−1.80	1.9442	H → L (61%), H − 1 → L (29%)	0.989
**TN+1-a**	−0.55	−0.68	−1.51	1.9640	H → L (59%), H − 1 → L (31%)	0.989
**TN+2-a**	−0.61	−0.43	−1.26	1.7537	H → L (56%), H − 1 → L (31%)	0.982
**T2N+1-a**	−0.57	−0.40	−1.23	1.7036	H → L (63%), H − 1 → L (28%)	0.980
**T2N+2-a**	−0.57	−0.76	−1.59	1.7718	H → L (45%), H − 1 → L (32%)	0.983
**TN-s**	−0.36	−1.09	−1.92	2.2651	H → L (69%), H − 1 → L (21%)	0.995
**TN+1-s**	−0.56	−0.88	−1.71	2.3316	H → L (64%), H − 1 → L (23%)	0.995
**TN+2-s**	−0.80	−0.87	−1.70	2.4503	H → L (58%), H − 1 → L (28%)	0.996
**T2N+1-s**	−0.68	−0.82	−1.65	2.5318	H → L (77%), H − 1 → L (9%)	0.997
**T2N+2-s**	−0.57	−0.76	−1.59	1.8501	H → L (43%), H − 1 → L (50%)	0.986

^a^ ∆*G*_inj_ = E_HOMO_ − E_VB_(NiO) [3,29]; ^b^ ∆*G*_reg_ = E(I^−^/I_3_^−^) − E_LUMO_ [3,29]; ^c^ ∆*G*_CR_ = E_LUMO_ − E_VB_(NiO) [3,29].

**Table 3:** Adsorption energy (*E*_Ad_) and bond lengths between the dye and (NiO) _10__×__2_.

Dyes	*E*_Ad_ kcal/mol	Band Length (Å)		Dyes	*E*_Ad_ kcal/mol	Band Length (Å)	
		Ni1-O1	Ni2-O2			Ni1-O1	Ni2-O2
**T1**	−37.88	2.05	2.06	**TN+1-a**	−41.82	2.02	2.06
**T2**	−38.72	2.03	2.06	**TN+2-a**	−42.32	2.05	2.06
**T3**	−42.92	2.06	2.07	**T2N+1-a**	−42.49	2.05	2.06
**TN-d**	−39.65	2.04	2.06	**T2N+2-a**	−37.14	1.99	1.99
**TN+1-d**	−42.28	2.05	2.06	**TN-s**	−42.79	2.06	2.07
**TN+2-d**	−42.96	2.02	2.06	**TN+1-s**	−43.83	2.04	2.06
**T2N+1−d**	−44.33	2.05	2.07	**TN+2-s**	−44.94	2.03	2.06
**T2N+2-d**	−41.25	2.03	2.06	**T2N+1-s**	−45.22	2.04	2.05
**TN-a**	−39.16	2.05	2.06	**T2N+2-s**	−43.35	2.05	2.06

## Supplementary Materials

The following are available online at https://www.mdpi.com/1420-3049/24/17/3134/s1.
