# Supplementary material for: Effect of Nitrogen Cation as “Electron Trap” at π-Linker on Properties for p-Type Photosensitizers: DFT Study
Source: Molecules. 2019 Aug 28;24(17):3134. doi: 10.3390/molecules24173134 (PMC6749191; doi:10.3390/molecules24173134)
Supplement: Supplementary file 1 [file molecules-24-03134-s001.pdf]

Supporting Information

Effect of nitrogen cation as "electron trap" at π-linker on properties for *p*-type photosensitizers: DFT study

Zhi-Dan Sun [1], Jiang-Shan Zhao [1], Xue-Hai Ju [1,*], Qi-Ying Xia [2,*]

[1] Key Laboratory of Soft Chemistry and Functional Materials of MOE, School of Chemical Engineering, Nanjing University of Science and Technology, 210094 Nanjing, P. R. China; zdsun163@163.com (Z.-D.S); jszhao@njust.edu.cn (J.-S.Z)

[2] School of Chemistry and Chemical Engineering, Linyi University, 276005 Linyi, P. R. China

* Correspondence: xhju@njust.edu.cn (X.-H.J.); xiaqiying@163.com (Q.-Y.X.)

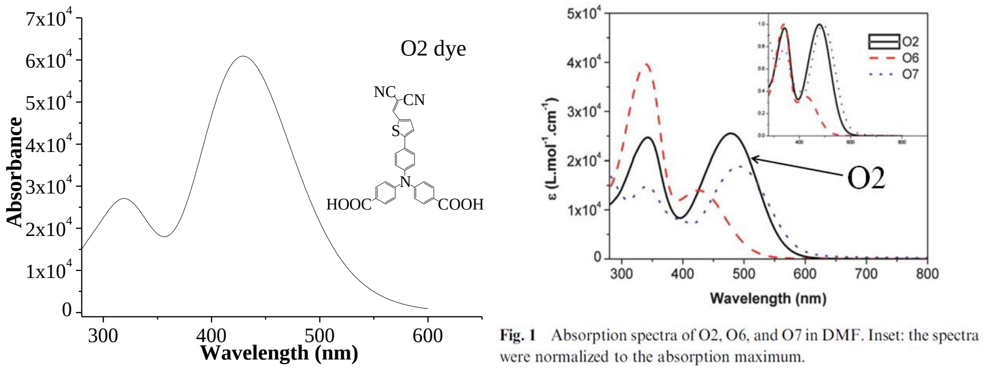

Figure S1. Absorption spectrum of O2 dye calculated at CAM-B3LYP/ 6-311G** level, as well as its experimental spectrum [4]

Table S1. The absorption width and onset at half maximum of the strongest absorption peak of the UV-vis absorption spectra

Dyes	Full width at half maximum of the strongest absorption peak (nm)	Onset of full width at half maximum of the strongest absorption peak (nm)	Dyes	Full width at half maximum of the strongest absorption peak (nm)	Onset of full width at half maximum of the strongest absorption peak (nm)
**T1**	118.6	415.4	**T2**	128.0	425.1
**T3**	134.2	433.9	**TN-d**	127.2	420.7
**TN+1-d**	125.7	426.9	**TN+2-d**	109.8	398
**T2N+1-d**	128.4	431.5	**T2N+2-d**	102.1	373
**TN-a**	122.9	414.7	**TN+1-a**	130.8	433.7
**TN+2-a**	114.7	404.4	**T2N+1-a**	142.1	448.5
**T2N+2-a**	98.4	384.2	**TN-s**	130.0	430.5
**TN+1-s**	128.9	426.9	**TN+2-s**	116.2	403.1
**T2N+1-s**	136.3	437.6	**T2N+2-s**	108.4	390.6
